# Adoptive T-cell therapy for Leukemia

**DOI:** 10.1186/2052-8426-2-25

**Published:** 2014-08-12

**Authors:** Haven R Garber, Asma Mirza, Elizabeth A Mittendorf, Gheath Alatrash

**Affiliations:** Department of Stem Cell Transplantation and Cellular Therapy, University of Texas MD Anderson Cancer Center Houston, Houston, 77030 Texas; Department Surgical Oncology, University of Texas M.D. Anderson Cancer Center, Houston, Texas

**Keywords:** Immunotherapy, Adoptive cellular therapy, T-cell, Stem cell transplant, Leukemia, Chimeric antigen receptor, Engineered T-cell, Tumor antigen

## Abstract

Allogeneic stem cell transplantation (alloSCT) is the most robust form of adoptive cellular therapy (ACT) and has been tremendously effective in the treatment of leukemia. It is one of the original forms of cancer immunotherapy and illustrates that lymphocytes can specifically recognize and eliminate aberrant, malignant cells. However, because of the high morbidity and mortality that is associated with alloSCT including graft-versus-host disease (GvHD), refining the anti-leukemia immunity of alloSCT to target distinct antigens that mediate the graft-versus-leukemia (GvL) effect could transform our approach to treating leukemia, and possibly other hematologic malignancies. Over the past few decades, many leukemia antigens have been discovered that can separate malignant cells from normal host cells and render them vulnerable targets. In concert, the field of T-cell engineering has matured to enable transfer of ectopic high-affinity antigen receptors into host or donor cells with greater efficiency and potency. Many preclinical studies have demonstrated that engineered and conventional T-cells can mediate lysis and eradication of leukemia via one or more leukemia antigen targets. This evidence now serves as a foundation for clinical trials that aim to cure leukemia using T-cells. The recent clinical success of anti-CD19 chimeric antigen receptor (CAR) cells for treating patients with acute lymphoblastic leukemia and chronic lymphocytic leukemia displays the potential of this new therapeutic modality. In this review, we discuss some of the most promising leukemia antigens and the novel strategies that have been implemented for adoptive cellular immunotherapy of lymphoid and myeloid leukemias. It is important to summarize the data for ACT of leukemia for physicians in-training and in practice and for investigators who work in this and related fields as there are recent discoveries already being translated to the patient setting and numerous accruing clinical trials. We primarily focus on ACT that has been used in the clinical setting or that is currently undergoing preclinical testing with a foreseeable clinical endpoint.

## Introduction

T-lymphocytes are critical cells of the immune system that eliminate both infectious pathogens and abnormal, malignant cells. The importance of T-cells in the elimination of leukemia was first supported by trials comparing T-cell replete versus T-cell depleted allogeneic stem cell transplant (alloSCT), where a higher incidence of disease relapse was observed for recipients of T-cell depleted transplants, but with the advantage of a lower incidence of graft-versus-host disease (GvHD) [1–6]. The data for this effect are strongest in chronic myeloid leukemia (CML), though evidence for T-lymphocyte targeting of malignant cells within other leukemia diagnoses – acute myeloid leukemia (AML), chronic lymphocytic leukemia (CLL), and acute lymphoblastic leukemia (ALL) - also comes from trials demonstrating success with donor lymphocyte infusion (DLI) for relapsed leukemia after alloSCT [7–11]. DLI is a treatment comprised of unselected, polyclonal donor lymphocytes and is frequently administered in the absence of preparative conditioning. It provides the most direct proof of the potent graft-versus-leukemia (GvL) effect mediated by allogeneic T-cells, but GvHD complicates DLI in up to 60% of patients [12]. Thus, adoptive cellular therapy (ACT) has arisen from the desire to isolate and enhance the GvL effect that accounts for the efficacy of alloSCT and DLI while minimizing GvHD toxicity, which causes significant morbidity and mortality. ACT is a type of cancer treatment that involves infusing patients with large numbers of autologous or allogeneic lymphocytes that have undergone *ex vivo* selection and modification. The goal of ACT for leukemia is to administer T-cells that target leukemia antigens with minimal impact on normal tissues.

It is important to highlight that GvL and GvHD both refer to the allogeneic setting where donor T-cells are presumed to recognize both tumor-associated antigens (nonpolymorphic self antigens that are overexpressed in malignant cells), minor histocompatibility antigens (polymorphic host antigens that are foreign to the donor) and tumor-specific antigens (antigens that are mutated or solely expressed by the tumor cell) [13, 14]. Graft-versus-tumor effects are not exclusive to allogeneic T-cells, however, and Rosenberg et al. have pioneered efforts to use a patient’s autologous T-cells to combat melanoma, and more recently carcinoma, using several strategies with much success [15, 16]. With regard to hematologic disease, using ACT is a natural extension of standard of care approaches that are currently employed to treat leukemia, lymphoma, and myeloma - specifically autologous and alloSCT. Limiting this approach, though, are a lack of known tumor antigens and mechanisms of central and peripheral T-cell tolerance whereby T-cells with high affinity for self-antigens are deleted in the thymus or are rendered hyporesponsive through various mechanisms that can be exploited by the immunosuppressive tumor microenvironment [17]. Numerous high throughput methodologies are being explored for the identification of novel tumor antigens, and, to bypass T-cell tolerance, research is now capitalizing on advances made in synthetic biology and basic immunology to engineer and redirect T-cells to eliminate tumor cells. The purpose of this review is to provide an overview of various strategies being developed to improve the adoptive transfer of T-cells for immunotherapy of leukemia, with a focus on the approaches being tested in clinical trials.

## Review

### Leukemia antigens

Arguably, the most important aspect of ACT is the targeted antigen, and this is becoming increasingly true as methods to enhance the T-cell receptor (TCR) affinity and to lower T-cell activation thresholds are incorporated. These improvements narrow the therapeutic window for ACT and necessitate careful antigen selection. Many, but not all, tumor antigens arise from intracellular proteins that must be processed and presented by a cell’s major histocompatibility complex (MHC) in order to trigger TCR-binding and provoke an immune response. In contrast, the implementation of chimeric antigen receptors (CARs) has now broadened the pool of potential antigens to include extracellular, non-MHC bound molecules. The ideal tumor antigen is expressed on all malignant cells including cancer stem cells, demonstrates high immunogenicity, is absent in normal tissue, and derives from a protein required for maintenance of the malignant phenotype, which prevents a leukemic subclone from escaping T-cell detection by downregulating the antigen’s expression [18].

There are several classes of tumor antigens (Table 1). Many are tumor-associated antigens (TAAs) derived from self-proteins that are expressed at low levels in normal tissue and overexpressed or aberrantly expressed in malignant cells, such as Wilms tumor-1 (WT-1) [19]. Tumor differentiation antigens are a subset of TAAs that arise from self-proteins and are expressed in a distinct subset of normal cells and often at higher levels in their malignant cell counterpart. PR1, a peptide from neutrophil elastase and proteinase 3, is an example of a tumor differentiation antigen as its source proteins are induced during the promyelocyte stage and are overexpressed in myeloid leukemias [20–22]. Targeting these classes of self-antigens has shown promise in preclinical models, vaccine trials, and early adoptive T-cell trials, but risks toxicity to normal tissues as the antigens are not strictly found on malignant cells. This is also true for the cancer-testis antigens, a family of proteins expressed primarily by germ cells and in various malignancies [23]. Several recent trials highlight the risks of targeting TAAs, including a trial at the National Institutes of Health (NIH) that tested the adoptive transfer of autologous T-cells engineered to express a murine TCR with specificity for an HLA-A2-restricted peptide from carcinoembryonic antigen (CEA) [24]. Anti-CEA T-cells were administered to 3 patients with metastatic colon cancer. The treatment caused tumor regression, including 1 partial response (PR), but severe, dose-limiting toxicity in the form of inflammatory colitis was observed in all patients and caused the trial to be halted. The colitis was an on-target adverse effect resulting from T-cells recognizing the CEA epitope on normal colonic epithelium, illustrating that the targeting of self-antigens with modified T-cells is not without consequence.

**Table 1:** **Classes of Leukemia Antigens**

Leukemia antigens				
Class	Example(s)	Pros	Cons	
**Tumor-associated antigens (TAAs)**	WT-1 [25], hTERT [26], PRAME [27], HMMR/Rhamm [28]	- multiple candidates identified- often shared by > 1 malignancy	- present on normal tissue	**- self antigens that generate low-avidity T-cells- must be successfully processed and presented by the MHC of the malignant cell**
**Tumor differentiation antigens**	PR1 [29], CG1 [30], CD33 [31]	- more restricted distribution than TAAs	- present on subset of normal cells, which can include hematopoietic stem cells	
**Cancer testis (CT) antigens**	Cyclin-A1 [32], NY-ESO-1 [33] , MAGE [34]	- frequently restricted to non-essential tissues and tumor	- few identified in leukemia	
**Minor histocompatibility antigens (mHAs)**	HA-1 [35], ACC1 [36], T4A [37], LB-LY75-IK [38]	- result in high avidity allo T-cells since epitopes are foreign to donor- some are largely restricted to hematopoietic compartment	- Necessitate rescue with mHA-negative stem cells to restore normal hematopoiesis- need for allogeneic TCRs	**- must be successfully processed and presented by the MHC**
**Tumor-specific antigens (neoantigens)**	BCR-ABL [39], FLT3-ITD [40], B-cell receptor idiotype [41]	- result in high avidity autologous T-cells- many derive from proteins critical in leukomogenesis	- individual-specific- few identified in leukemia since mutation rate is low	
**Oncoviral antigens**	HTLV-I Tax protein [42]	- generate very high-avidity T-cells	- only relevant to virus-initiated malignancies	
**Extracellular antigens**	CD19 (see CD19 section), Lewis Y [43], CD22 [44], ROR1 [45]	**-MHC-independent**- interaction with CAR is high-affinity	- many are present on normal tissues	- **require CAR for targeting**, which can mediate on-target, off-tumor adverse effects

Additional references added in the table [31–34, 36–38, 40, 45].

In the allogeneic setting, minor histocompatibility antigens (mHAs) are endogenous polymorphic proteins that differ between donor and recipient and can serve as targets for GvL and GvHD [13]. These represent important antigens because donor cells are not tolerized to these ‘foreign’ peptides enabling them to elicit higher avidity T-cell responses. One goal is to identify mHAs that are confined to the hematopoietic compartment, because in this scenario, after the host blood system is replaced with donor cells by alloSCT, the only mHA-expressing cells remaining are malignant host cells. At least 12 such hematopoietic-predominant mHAs have been identified thus far and one such antigen, HA-1, has been targeted in a pilot clinical trial [35, 46].

Also in the category of "non-self" antigens are neoantigens, otherwise known as tumor-specific antigens. These are novel peptides that result from chromosomal translocations, point mutations, deletions, or insertions within a malignant cell’s genome. Neoantigens theoretically represent ideal targets as they should elicit high avidity T-cells, they are absent on normal cells, and they may originate from "driver" genes that are leukemogenic. In melanoma, a highly mutated disease, it is thought that neoantigens constitute much of the anti-tumor immune response [47–49]. Leukemias, however, are among cancers with the lowest mutation rate, making such antigens difficult to identify [50, 51]. In CML, the BCR-ABL translocation does represent one source for leukemic neoantigens, and several epitopes from this fusion protein have been shown to elicit a T-cell response [52–55]. Early trials of adoptively transferred anti-BCR-ABL T-cells have proven safe with some evidence of disease response, however, the success of tyrosine kinase inhibitors in CML has largely overshadowed the development of ACT for this disease [56, 57].

Tumor-specific antigens (neoantigens) can also be found within the clonal idiotypes unique to B-cell malignancies, which contain the variable regions of the B-cell receptor immunoglobulin heavy and light chains [58]. Vaccination strategies against these epitopes have demonstrated success in lymphoma [41]. These antigens are patient-specific, though, which raises questions about the feasibility of using these epitopes and other highly individualized neoantigens as targets. With advances in whole exome sequencing and T-cell engineering, though, they will likely gain importance.

The last class of "non-self" intracellular antigens are oncoviral peptides, which are being investigated as immunotherapeutic targets in human papillomavirus-initiated cervical cancer, Epstein Barr Virus (EBV)-related post-alloSCT B-cell lymphoproliferative disease, and EBV-associated lymphomas [59–61]. They may also be applicable in the setting of virally-induced hematologic malignancies, such as HTLV-1 associated T-cell leukemia/lymphoma [42, 62]. The advantage of these viral peptides is that they elicit very high avidity T-cell responses in contrast to endogenous human epitopes [63].

Finally, it is important to emphasize that all of the tumor antigens discussed thus far are intracellular antigens, which rely on intact, functional cellular machinery for their processing and MHC-restricted presentation. This is a fundamental limitation of TCR-based T-cell therapy because human leukocyte antigen (HLA) subtypes are highly polymorphic and labor-intensive strategies that target a single epitope will only apply to the subset of patients expressing that particular HLA allele. Furthermore, malignant cells are known to evade the immune system by altering antigen processing and downregulating their MHC expression [64]. One strategy to bypass these constraints is through use of CAR T-cells that target broadly expressed membrane-bound molecules. A CAR links a portion of the antibody binding domain to T-cell costimulatory and signaling domains [65]. Because the recognition domain is engineered from an immunoglobulin, the CAR recognizes extracellular, membrane-bound epitopes that are not confined to an MHC, and this groundbreaking development has added a new class of leukemia antigens and vastly expanded the number of targetable epitopes. Dozens of extracellular molecules are now being investigated for their value as tumor antigens and, in leukemia, these include CD19, CD20, CD22, CD30, Lewis Y antigen, among others [66]. However, although the immune response can be effectively engineered using this technology, the antigens that CAR T-cells target must still be evaluated for their expression in normal tissues and for their importance in leukemogenesis to limit the possibility of selecting for antigen escape variants. Lastly, even though the majority of CAR T-cells developed to date have targeted broadly expressed cell surface receptors, CAR T-cells that target peptides presented on HLA molecules are also currently being investigated and work by integrating TCR-mimicking antibodies [67]. It is likely that successful immunotherapy for leukemia will require targeting many of these antigens in multiple pathways, which likely accounts for the success of the graft-versus-leukemia effect.

### Adoptive T-cell Transfer Strategies for Leukemia

Although this review focuses specifically on modifications and *ex vivo* manipulation of CD4+ and CD8+ αβ T-lymphocytes for adoptive transfer and treatment of leukemia, ACT using other effector cell types – including γδ T-cells, natural killer (NK) cells, NK T-cells, and invariant NK T cells - is also an area of active investigation [68–71]. Adoptive T-cell therapy represents a natural extension of current treatment for leukemia because allogeneic transplant and DLI already involve the transfer of large numbers of T lymphocytes. Further, adoptive T-cell transfer to prevent CMV, adenovirus, and Aspergillus infections in the context of alloSCT has been well studied and provides a valuable foundation for T-cell transfer strategies [72–78]. T-cells have many properties of an ideal therapeutic - their area of biodistribution is broad and includes the central nervous system, they can expand and increase their potency *in vivo*, and they can acquire long half-lives by establishing memory, ideally providing a reservoir of antitumor immunity that leads to actual cancer cures [79, 80].

**Figure 1:**
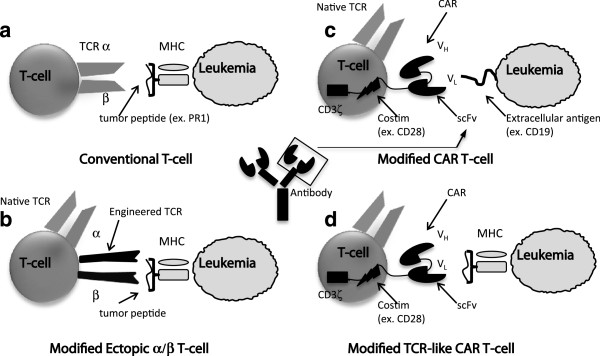
**Graphic representations of the various T-cell approaches used to target leukemia antigens. (a)** Conventional T-cells target peptides that are presented in the context of an MHC molecule. **(b)** Modified Ectopic α/β T-cells target the same epitope as a conventional TCR (MHC-peptide) but with an engineered (often higher affinity) TCR from an autologous, allogeneic, or xenogeneic cell. **(c)** CAR T-cells contain a synthetic polypeptide that contains the single chain variable fragment (scFv) of an antibody as the antigen-binding domain, a hinge, a transmembrane region, a costimulatory (Costim) domain, and a CD3ζ signaling domain. CARs recognize extracellular antigens that are not MHC-bound. **(d)** A TCR-like CAR utilizes the scFv fragment of a TCR-mimicking Ab, which recognizes a peptide antigen in the context of an MHC molecule, but with a much higher affinity than conventional or most engineered TCRs.

There are numerous strategies being developed to target malignant cells via adoptive T-cell transfer (Figure 1). A major division within current ACT approaches is whether or not T-cells undergo genetic modification prior to infusion. T-cells that do not undergo this additional step (conventional T-cells) must undergo *ex vivo* manipulation and expansion prior to administration, an extensive process where cells may spend a month or more in culture. The goal of this step is to generate large numbers of T-cells that are specific for one or more tumor antigens from an infrequent and polyclonal population. In contrast, genetic modification of T-cells bypasses these lengthy enrichment protocols by direct transfer of either TCR α and β chains or a CAR, thereby endowing lymphocytes with a secondary, engineered specificity. The rationale, advantages, and disadvantages for the most common strategies are presented here.

Transfer of unmodified, conventional T-cells includes both autologous and allogeneic lymphocytes that range in antigen specificity from polyclonal populations to antigen-enriched subsets to antigen-specific T-cell clones. Transfer of allogeneic polyclonal T-cells describes a DLI, which can be effective for treating relapsed leukemia after alloSCT though with a high risk of GvHD [12]. Adoptive transfer of bulk, *ex vivo* activated, autologous polyclonal lymphocytes has been tested by Rapoport et al. and, when administered with tumor vaccines, can generate antitumor immunity [81]. However, both central and peripheral tolerance hinder this approach and T-cells with specificity for tumor antigens are infrequent, have low avidity, and can be rendered anergic by the immunosuppressive tumor microenvironment [17, 63]. A number of tumor-specific T-cell enrichment strategies exist and include culturing lymphocytes in the presence of irradiated tumor cells, stimulating mHA-naive allogeneic T-cells in the presence of mHA-positive host antigen-presenting cells (APCs), or extracting T-cells from tumor specimens and subsequently expanding them for reinfusion. For example, a recent trial at the NIH tested the adoptive transfer of polyclonal tumor-derived lymphocytes for the treatment of relapsed CLL or lymphoma after alloSCT [82]. The rationale underlying this approach is that T-cells found within tumor specimens are enriched for tumor antigen-specific cytotoxic T-lymphocytes (CTLs), which can be made more potent by *ex vivo* costimulation and expansion. This is analogous to tumor-infiltrating lymphocyte (TIL) therapy for melanoma, which has demonstrated overall response rates of up to 72% [83]. In the lymphoma/CLL trial, 8 patients were treated with lymphocytes that were first extracted from their tumor biopsy specimens and subsequently activated and expanded with anti-CD3/anti-CD28 Ab-coated magnetic beads. No GvHD was observed and modest anti-tumor activity was seen in 4 patients [82]. Additionally, towards the goal of targeting multiple antigens simultaneously, a recent study demonstrated that autologous, functional T cells from ALL patients could be generated with specificities enriched for multiple TAA (PRAME, WT-1, MAGE-A3, and Survivin) by stimulating lymphocytes using autologous APCs pulsed with complete peptide pools spanning the entire amino acid sequences of the tumor-associated proteins [84].

The most precise approach using conventional T-cells is transfer of antigen-specific CTL clones, which are generated by labor-intensive limiting dilution cloning techniques [85]. These methods produce large numbers of monoclonal T-cells that recognize a defined HLA-restricted tumor epitope. Autologous or allogeneic CTL clones can be generated using a variety of stimulator cells. For example, large numbers of CTL clones with specificity for an immunodominant HLA-A2-restricted epitope from WT-1 were generated by stimulating donor T-cells repeatedly with peptide-pulsed autologous DCs for use in a recent clinical trial (discussed below) [25]. There are benefits to using conventional T-cells over T-cells genetically engineered to express an ectopic TCR α and β chain. With conventional T-cells, there are no concerns for ectopic and endogenous TCR α/β mispairing, which has the potential to generate new specificities and cause harm by nonspecifically reacting with normal tissues [86]. Also, though viral transduction of TCRs into mature cells has thus far proven safe, the use of conventional T-cells also eliminates the worry for insertional oncogenesis of transduced lymphocytes [87, 88]. Additionally, with conventional allogeneic CTL clones, there is no secondary specificity of the T-cells and thus a much lower risk for off-target GvHD. Finally, conventional T-cells – whether autologous or allogeneic – have been educated in the human thymus and have toxicities that are more predictable than those for murine TCRs and affinity-enhanced TCRs, which have exhibited deleterious off-target effects [89]. On the other hand, the efficacy of adoptive transfer of conventional T-cells is limited by several factors. Tumor antigen-specific T-cells, particularly those that recognize "self" antigens, are generally of low avidity - a problem that can be exacerbated in patients with leukemia [90]. Moreover, generating large numbers of clones requires weeks of repetitive stimulation in culture, which can further reduce T-cell avidity while allowing time for tumors to progress. Similarly, extensive *ex vivo* manipulation can select for T-cells with more differentiated, exhausted effector phenotypes that are less likely to mediate durable, potent tumor regression after adoptive transfer [91].

To bypass many of these limitations, methods have been developed to deliver the genes for full-length TCR α and β chains into autologous or allogeneic peripheral blood mononuclear cells (PBMC), creating bispecific T-cells with specificities determined by both the cell’s endogenous TCR and the engineered TCR [92]. This approach dramatically shortens the *ex vivo* manipulation process and allows for generation of high avidity clones. This strategy also allows for pre-selection of a desired subset of lymphocytes for TCR transduction (central memory T-cells, stem cell memory T-cells, etc.), which have demonstrated better persistence and expansion *in vivo* relative to the terminally differentiated, effector lymphocytes often generated by lengthy limiting dilution cloning protocols [93–96]. Retroviruses, lentiviruses, transposons, and other constructs are all being actively investigated as means to deliver the TCR (or CAR – see below), and the pros and cons of each of these methods are outside the scope of this discussion but have been reviewed by others [97, 98]. Many refinements to TCR-engineering protocols have already been developed and include the addition of disulfide bonds to prevent TCR α/β mispairing and codon optimization to facilitate better TCR expression [99, 100]. Additionally, very high affinity xenogeneic TCRs from HLA-A2 transgenic mice can be transferred into human PBMC using these methods [101–104]. TCR affinity can be further enhanced via site-directed mutagenesis of the TCR antigen-binding region [105]. Unfortunately, some of these "supercharged" T-cells have demonstrated unpredictable toxicities in patients, and new safety measures for testing high-affinity, modified T-cells against human tissue samples prior to their use in clinical trials will need to be implemented [89, 106, 107]. Nevertheless, several TCR constructs derived from conventional, human T-cells with physiologic avidities have undergone extensive preclinical testing and will now be studied in human leukemia trials including those that redirect T-cells against HLA-A2 restricted epitopes of HA-1 and WT-1 [100, 108]. One potential drawback to all types of TCR-based ACT is that the binding between the TCR and MHC/peptide complex is inherently degenerate, meaning that a given TCR can recognize more than 1 peptide-MHC ligand [109]. This could result in unforeseen toxicity in the form of off-target tissue effects, particularly when large numbers of cells with affinity enhanced or xenogeneic TCRs are infused. Lastly, though ectopic TCR transfer overcomes many of the limitations posed by conventional T-cells, the targeted epitopes must still be successfully processed within the proteasome and subsequently presented in the context of an MHC molecule, which may afford malignant cells the opportunity to escape this therapy via downregulation of surface MHC or aberrant antigen processing.

As alluded to above, CARs represent a unique strategy designed to circumvent many drawbacks of TCR-based ACT. The antigen-recognition domain of the CAR is derived from an antibody’s single-chain variable fragment (scFv), which recognizes epitopes within membrane-bound molecules. The majority of CARs developed to date target broadly expressed surface molecules that are independent of MHC, though CARs can be engineered to recognize peptide-HLA epitopes as well [67]. The scFv fragment is linked to a transmembrane region, a signaling protein (typically CD3ζ), and a costimulatory domain (CD28, 4-1BB, OX-40, ICOS, and others). The CARs are delivered using vehicles similar to those discussed above for ectopic TCRs (lentiviruses, retroviruses, etc.). CARs were first reported by Gross et al. in 1989 [65]. Early clinical trials using CARs demonstrated disappointing efficacy, largely due to the lack of persistence of the modified cells [110–112]. These 1^st^ generation CARs lacked a costimulatory domain. The addition of a costimulatory domain (initially CD28 or 4-1BB) has revolutionized the field and these 2^nd^ generation CARs have begun to demonstrate impressive clinical responses, particularly using anti-CD19 CARs for treatment of B-cell malignancies (see CD19 section below). Now, 3^rd^ generation CARs (those with >1 engineered costimulatory domain) are reaching clinical testing and may further enhance the redirected T-cells’ activity. By targeting extracellular epitopes, CARs have the distinct advantage of thwarting many mechanisms of tumor immunoevasion including MHC downregulation and altered protein processing. The problem of tumor escape is not solved completely, however, and malignant cells with little or no expression of the targeted antigen may evade this approach, underscoring the importance of targeting proteins that are critical in leukemogenesis [79]. Another important advantage of CARs is that they are very high-affinity receptors that mediate eradication of malignant cells with low levels of antigen expression. A consequence of this enhanced affinity is that normal cells with low antigen expression will also be targeted resulting in increased on-target, off-tissue adverse effects [24, 113, 114]. CAR and ectopic TCR approaches share many of the same advantages over conventional T-cell ACT. For example, the subset of T-cells to be transduced with either artificial receptor can be pre-enriched for central memory T-cells, stem cell memory T-cells, or virus-specific T-cells and either allogeneic or autologous cells can be used for transduction. A fundamental difference between CARs and ectopic TCRs is that they target extracellular or intracellular antigens, respectively, but even this line is being blurred. TCR-mimicking antibodies against PR1 and WT-1 have been generated and are now being tested in TCR-like CAR constructs in preclinical mouse models [67, 115, 116]. These receptors function like TCRs since they recognize a peptide antigen in the context of an MHC molecule, but they have the benefit of binding with higher affinity than a TCR. These and other recent advances illustrate how the field of ACT is rapidly evolving, and it is likely that genetically modified T-cells will overtake conventional T-cells within the field of ACT.

### Additional T-cell modifications

Specificity is not the only focus of T-cell engineering. Though outside the scope of this review, other authors have outlined the many ways by which researchers are aiming to build a better T-cell [66, 97, 117–119]. Examples of these strategies include incorporating additional chemokines to improve T-cell trafficking, artificially upregulating anti-apoptotic proteins, increasing resistance to immunosuppressive cytokines, and adding suicide genes to delete the modified lymphocytes in the event of significant toxicity [120–126].

### Allogeneic SCT platform

**Figure 2:**
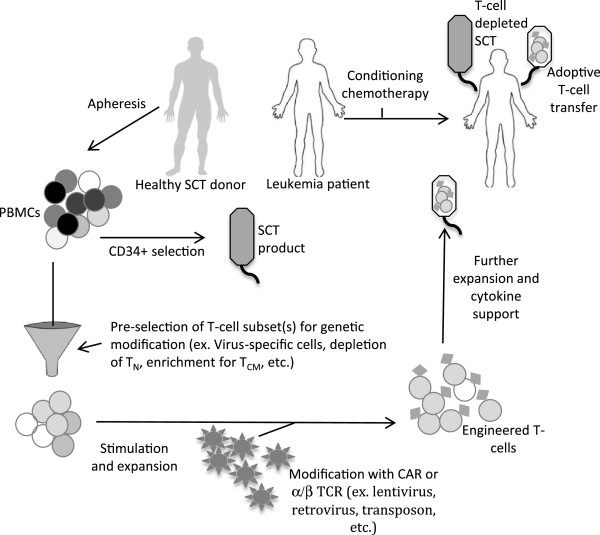
**Diagram of ACT within the alloSCT platform.** T-cell depleted SCT would eliminate the need for post-SCT immunosuppressive medications that can limit the efficacy of modified T-cells. Since allogeneic cells are used, the endogenous specificity of the T-cells would be pre-selected (CMV, EBV, varicella, etc.) or other GvHD prevention measures would need to be incorporated, such as naïve T cell depletion.

In many ways, ACT for leukemia lends itself to the allogeneic SCT setting where large numbers of allogeneic T-cells are infused along with CD34+ progenitor cells after conditioning chemotherapy. ACT is not limited to this setting, however, and anti-CD19 CAR therapy in the absence of transplant and in the autologous setting has demonstrated success in heavily pretreated and refractory CLL patients [80]. Many questions will need to be answered to utilize ACT most effectively and to properly position it alongside current treatments including cytotoxic chemotherapy, molecular-targeted agents, and alloSCT (Figure 2). Cost and manufacturing capabilities will undoubtedly factor into these decisions as well. It is conceivable that ACT could become initial, first-line therapy for certain leukemia subtypes in contrast to the challenging, post-alloSCT relapse setting where it is typically tested today. Moreover, progress continues to be made in other types of therapy making treatment decisions a moving target. For example, alloSCT has historically been reserved for young, healthy patients with relapsed or high-risk disease, but this is changing with the advent of nonmyeloablative transplant and improved supportive care [127, 128].

One aspect of ACT that is established and relevant to leukemia is the benefit achieved with lymphodepletion. This has been studied in the non-transplant setting where depleting host lymphocytes prior to adoptive T-cell transfer improves T-cell expansion and persistence by freeing up important cytokines (IL-7, IL-15), encouraging homeostatic peripheral expansion, and depleting regulatory T-cells [129–133]. Conditioning chemotherapy prior to alloSCT would then serve a dual purpose in this setting – eradication of disease and creation of space for transferred leukemia-specific T-cells. Additionally, it may be advantageous for patients to be in a state of minimal residual disease (MRD) at the time of ACT, which is often the situation after alloSCT. This may be especially true for aggressive malignancies like AML and ALL, where the leukemia burden can quickly increase. The reasons for this are two-fold: first, it has been observed in early clinical trials that ACT-associated toxicities such as the cytokine-release syndrome are more severe in patients with larger disease burdens and second, it can take days to weeks for modified T-cells to expand *in vivo* and so an MRD state affords the modified T-cells time to proliferate and potentially overtake the malignant cell population. Unfortunately, it is not always possible for patients to reach a state of MRD, and early trials have shown that anti-CD19 CAR therapy can mediate dramatic leukemia responses even in patients with very advanced disease, proving its versatility [79, 134, 135].

Two main concerns will need to be addressed to successfully apply ACT to the alloSCT setting: the potential for triggering GvHD and interference caused by immunosuppressive medications. Since the recipient’s hematopoietic system is replaced by donor cells after alloSCT, conventional or modified adoptively transferred T-lymphocytes will need to be of donor origin. This being said, in the future it may be possible to use universal or 3^rd^ party donor T-cells by incorporating zinc-finger nuclease technology that can be used to knockdown a T-cell’s MHC class I/II expression as well as its endogenous TCR [136]. Nevertheless, it is well established that infusing large numbers of allogeneic T-cells into patients shortly after transplant results in unacceptable rates and severity of GvHD [137]. Therefore, it will not be possible to simply transduce bulk donor T-cells with the desired antigen-specific TCR or CAR construct and infuse these cells at the time of transplant since the endogenous allogeneic polyclonal TCRs would trigger GvHD. This problem can be approached from many angles. One possibility is to isolate donor lymphocytes with defined, known specificities (EBV, CMV, varicella, etc.) and to subsequently transduce only these oligoclonal T-cells with a synthetic receptor (CAR or ectopic TCR) and then infuse them at the time of transplant [138, 139]. Another option is to deplete naïve donor T-cells prior to *ex vivo* TCR/CAR transduction since naive T-cells have been shown to play an important role in GvHD [140–142]. In contrast, if bulk polyclonal donor cells are used, delayed, low-dose administration of ACT at time of relapse or many months after alloSCT may mitigate GvHD. This is based on trials of DLI, where administration of low dose donor T-cells more than 9 months after transplant resulted in a GvHD rate of only 10% [143, 144]. Encouragingly and along these same lines, in early trials of anti-CD19 CAR therapy, infusions of modified, polyclonal donor-derived cells that were collected from the recipient (post-transplant) did not cause GvHD, possibly because of tolerance developed within the host [145, 146].

A separate platform for ACT that should be considered is T-cell depleted alloSCT. Though opportunistic infections would be expected to increase, this approach might be advantageous because it would eliminate the need for immunosuppressive agents, which are known to abrogate the effects of adoptively transferred T-cells [134, 135]. Otherwise, for T-cell replete alloSCT, an attractive strategy is to arm modified, antigen-specific T-cells with resistance to immunosuppressive drugs (calcineurin inhibitors, mycophenolate mofetil, methotrexate, or glucocorticoids) [46, 147–149]. Still, it must be kept in mind that the rationale for using allogeneic rather than autologous transplants for leukemia is both to avoid infusion of malignant cells and to harness the GvL effect. If the GvL effect is successfully condensed to a handful of tumor antigens, then tumor-restricted, antigen-specific TCR and/or CAR constructs could be developed to target these antigens. This would permit the use of autologous grafts and autologous ACT (minor histocompatibility antigens excluded), which could significantly reduce (if not eliminate) GvHD. As outlined, the rapid pace of discovery in the field of ACT is exciting. Developing the most effective ACT will require careful consideration of the optimal antigens to target (likely in combination), the T-cell approach to utilize, and which of the myriad additional modifications to incorporate into the engineered lymphocytes.

### Challenges and limitations

Though immunotherapy is typically characterized as less toxic and more specific than chemotherapy and other cancer therapies, multiple serious adverse effects in early clinical trials using ACT underscore its tremendous potency and risks. Adverse effects have resulted from multiple mechanisms. On-target, off-tissue toxicity describes the situation where a T-cell appropriately recognizes an antigen of interest (via TCR or CAR), but in the case where this antigen is also expressed on healthy tissues. One example of on-target, off-tissue toxicity was reported in a Phase I clinical trial of renal cell carcinoma patients treated with autologous CAR T-cells engineered to recognize an epitope within the carbonic anhydrase IX (CAIX) tumor-associated protein [113]. Multiple patients experienced significant liver toxicity due to the expression of CAIX on normal bile duct epithelium requiring cessation of treatment in 4 patients. This on-target, off-tissue toxicity can be mediated either by a CAR or a TCR, however, CAR T-cells may prove the worst offenders because of their inherent high affinity and built-in costimulation. Off-target toxicity occurs when the antigen receptor (CAR or TCR) recognizes a different epitope than is intended. This form of toxicity has been more common with TCRs, which are inherently degenerate, and particularly with mutated and xenogeneic TCRs that bypass education in the thymus and can have unpredictable specificities. For example, a patient-derived TCR with specificity for the cancer-testis antigen HLA-A*01-MAGE-A3 first underwent site-directed mutagenesis resulting in enhanced affinity of the receptor, was subsequently incorporated into a lentiviral vector, and was then used in a clinical trial testing the construct against myeloma and melanoma. Unfortunately, the first 2 patients treated experienced fatal cardiac toxicity that was incited by an off-target epitope within the normal cardiac myocyte protein titin (off-target titin peptide: ESDPIVAQY; on-target MAGE-A3 peptide: EVDPIGHLY) [89, 106]. These adverse events have since been carefully analyzed and have generated much discussion about strategies to predict on and off-target toxicity.

Issues of specificity and antigen selection are paramount to the success of ACT but other challenges remain. Even in the context of a highly avid T-cell and a tumor-restricted antigen, T-cell persistence, trafficking, and diminishing receptor transgene expression can all be problematic [150]. Furthermore, the tumor microenvironment is an inhospitable place for T-cells and adoptively transferred cells will need to resist inhibitory signals from tumor and stromal cells to remain effective [151]. Even after initial recognition and lysis of tumor cells, the adoptively transferred lymphocytes must then outcompete the tendency of malignant subclones to evade killing via downregulation of MHC molecules, altered antigen processing, and epigenomic and genomic evolution [64]. And on a broader scale, issues of cost, manufacturing, and standardization will also be important to the applicability of ACT and have been reviewed by others [97, 152]. Finally, even patients who experience durable, complete remissions from ACT can experience adverse effects in the form of cytokine release syndrome, tumor lysis syndrome, hemophagocytic lymphohistiocytosis, and B-cell aplasia though investigators and clinicians are learning a great deal about how to limit these toxicities. It must be kept in mind, though, that many of the patients treated with ACT thus far have dismal prognoses and limited to no treatment options in the absence of clinical trials. Furthermore, ACT is still in its infancy and if the current pace of innovation is sustained, it promises to grow in importance. Along these lines, below is a discussion of current strategies being tested in clinical trials.

### Targeting leukemia antigens in the clinic

#### CD19

Trials of anti-CD19 CAR T-cell therapy in patients with B-cell malignancies have generated exciting clinical results and underscore the tremendous potential of antigen-specific ACT for leukemia. These trials – spearheaded by groups at the NIH, Baylor College of Medicine, the University of Pennsylvania, and at Memorial Sloan Cancer Center (MSKCC) – have laid the foundation for this new therapeutic modality that is certain to gain importance in the treatment of ALL, CLL and B-cell lymphomas.

CD19 functions as a component of the B-cell co-receptor that enhances B-cell receptor signaling upon antigen encounter and CD19 expression is restricted to malignant and normal cells of the B-cell lineage [153, 154]. In numerous preclinical studies, anti-CD19 CAR T-cells demonstrated selective lysis of B-lineage tumor lines and primary leukemia/lymphoma cells *in vitro* and also showed potent antitumor activity in murine xenograft and immunocompetent mouse models [152, 155–164]. Subsequently in 2010, Kochenderfer et al. at the NIH reported the first successful use of anti-CD19 CAR T-cell treatment in a patient with advanced follicular lymphoma who received cyclophosphamide and fludarabine conditioning followed by a single infusion of autologous T-cells retrovirally transduced with a 2^nd^ generation CD19 CAR (CD28 costimulatory domain) along with IL-2 [165]. This patient experienced a dramatic partial response as well as B-cell aplasia in weeks 9 through 39 after CAR infusion, demonstrating the potency and CD19 specificity of this approach. In the expanded NIH cohort, which included 8 patients with lymphoma or CLL, 6 of 7 evaluable patients achieved objective disease responses, though in the context of concurrent cyclophosphamide/fludarabine chemotherapy [166]. Directly attributable to the CD19 CAR therapy, B-cell aplasia was observed in 4 of 8 patients for 6 months or more, demonstrating the persistence (and toxicity) of anti-CD19 CAR T-cells. Shortly thereafter, impressive responses for CLL patients were reported in a separate Phase I trial, where 3 patients were treated with a 2^nd^ generation anti-CD19 CAR that differed from the NIH construct in both the costimulatory domain (4-1BB in contrast to CD28) and the virus used for transduction (lentivirus as opposed to retrovirus) [80, 134]. Despite all 3 patients having extensive and chemorefractory disease at the time of CAR infusion, all 3 experienced objective responses – 2 complete responses (CRs)– and the CAR T-cells expanded *in vivo* more than 1000-fold and persisted beyond 6 months. A recent update for 14 heavily pretreated and refractory CLL patients treated on this protocol described an overall major response rate of 57% - including 3 patients with durable CRs that have lasted up to 35 months and 5 patients with partial responses (PRs) [167].

Encouraging results have also been demonstrated in both pediatric and adult acute lymphoblastic leukemia, a more aggressive malignancy that is less immune-susceptible and responds poorly to DLI. This is partly due to low expression of costimulatory molecules by ALL cells [8, 9, 168, 169]. One of the first CD19 CAR trials conducted in ALL patients was a Phase I study that used autologous T-cells retrovirally transduced to express an anti-CD19 CAR with a CD28 costimulatory protein. This trial enrolled 5 adult patients with relapsed disease [135]. Prior to CAR T-cell infusion, 2 patients had overt disease, 2 had MRD, and 1 was MRD negative. All patients underwent lymphodepletion with cyclophosphamide followed by a split-dose infusion of CAR-modified T-lymphocytes. All 4 patients with detectable disease had CRs and became MRD negative between days 8–59 after infusion, enabling eligible patients to receive allogeneic SCT. One patient with overt disease prior to CAR infusion relapsed with CD19+ disease 90 days after treatment, and all patients showed signs of normal CD19+ B-cell recovery, highlighting that CAR T-cells in these patients did not persist indefinitely in contrast to those in the aforementioned CLL trial that harbored the 4-1BB costimulatory domain. Importantly in this ALL trial, toxicity in the form of cytokine release syndrome was associated with a larger burden of disease at the time of CAR infusion, and the 2 patients with morphologic disease prior to treatment required high-dose steroid therapy for cytokine release syndrome, which likely abrogated the therapeutic effects of the CAR-modified lymphocytes. The initial promising results from this trial were recently updated to include responses for 16 adult patients with relapsed or refractory ALL who have been treated on this protocol [170]. The overall complete response rate to date is 88%. In those patients with gross morphologic disease prior to CAR infusion, 78% have experienced a CR, allowing many to receive alloSCT. In this trial, the benefit of treating the cytokine release syndrome with the IL-6 monoclonal antibody, tocilizumab, was demonstrated, and it was shown to relieve symptoms with a less profound impact on CAR T-cell expansion when compared to high-dose steroids.

Impressive response rates have similarly been observed in a separate ongoing Phase I trial for pediatric and adult relapsed ALL patients, which incorporates the identical CAR construct used in the above-referenced CLL trial (lentiviral transduction with 4-1BB costimulatory domain). Results were first reported for 2 pediatric patients who both demonstrated complete morphologic response to CAR therapy within 1 month despite recalcitrant disease, though 1 child relapsed 2 months later with CD19 negative lymphoblasts [79]. Both patients experienced significant toxicity, and the first child that was treated demonstrated severe but reversible cytokine release syndrome necessitating mechanical ventilation, vasopressor support, and aggressive medical therapy. Despite this, the tremendous expansion of anti-CD19 CAR T-cells to > 1000-fold *in vivo* and their persistence in 1 patient for >180 days is incredibly promising. Updates from this trial were also reported at a recent ASH meeting [145]. A total of 16 children and 4 adults with relapsed ALL have been treated and 82% of patients have experienced CRs - 3 patients are still pending evaluation. Unfortunately, 3 patients with an initial CR have since relapsed, reportedly with CD19+ disease. All patients in this trial experienced some degree of cytokine release syndrome and, as shown in previous trials, tocilizumab proved beneficial.

Importantly, 11 of the patients in this ALL trial had received prior allogeneic SCT [145]. For this cohort, the cells used to generate the anti-CD19 CAR treatment were collected from the recipient, however depending on the degree of chimerism at the time of pheresis, the majority of these cells were of donor origin. Encouragingly, GvHD was not observed in this group despite pre-treatment with cyclophosphamide, a phenomenon that could be attributable to the donor cells having acquired tolerance in the host as all patients were at least 6 months post-SCT [145]. In the alloSCT setting, investigators have already undertaken the next logical step, which is to determine whether allogeneic cells collected from healthy donors can be modified with an anti-CD19 CAR construct and used to treat leukemia patients. The advantage of this method is twofold: first, there is no leukemic contamination of the infused cells and, second, healthy donor lymphocytes are potentially more potent cytotoxic effectors since they have not been exposed to multiple rounds of chemotherapy and immunosuppressive agents. The primary concern with infusing large numbers of allogeneic cells is for GvHD, the most frequent complication of donor lymphocyte infusion (DLI), and thus investigators have proceeded cautiously with this approach [9, 171]. In one study, 8 patients with relapsed ALL or CLL were treated with healthy donor allogeneic T-cells that were first enriched for virus-specific lymphocytes (cytomegalovirus (CMV), EBV, or adenovirus) prior to their transduction with a 2^nd^ generation anti-CD19 CAR containing the CD28 costimulatory domain [138]. This approach is advantageous because the specificity of the endogenous TCR is for viral antigens and thus infusion of bispecific T-cells, those with endogenous TCRs specific for viral antigens and ectopic TCRs specific for CD19, poses less risk for GvHD. Furthermore, viral reactivation is common in the post-SCT setting and could expand modified, CAR-expressing T-cells through viral TCR-signaling and potentially enhance their antitumor effect. In this pilot trial, the bispecific T-cells persisted for a median of 8 weeks and resulted in 2 objective responses -1 CR for 3 months and 1 PR for 2 months. No GvHD was observed. As predicted, EBV reactivation in 2 patients resulted in a concomitant increase of CD19 CAR transgene expression.

Lastly, a recent trial at a separate center investigated the safety of treating relapsed post-SCT patients with anti-CD19-CAR T-cells collected from their healthy donors; however, in this trial the modified donor T-cells were not enriched for any particular endogenous specificity [146]. Ten patients with either relapsed CLL or lymphoma who had undergone prior alloSCT and at least 1 DLI were enrolled and underwent a single infusion of allogeneic CAR T-cells without any preparative lymphodepletion. Surprisingly, no GvHD was observed though CAR cells were largely undetectable after 1 month. Three patients experienced regressions of their malignancy, with 1 ongoing CR in a CLL patient at 9 months. The next planned trial of this allogeneic CD19 CAR-modified therapy will incorporate preparative lymphodepletion, which would be predicted to both increase the antitumor potency and potentially heighten the risk for GvHD.

Currently, there are 20 open trials listed on the clinicaltrials.gov website as of June 2014 that are studying anti-CD19 CAR therapy in various permutations. For example, one study is using autologous anti-CD19 CAR cells for children with relapsed ALL that incorporate an EGFRt tracking/suicide construct and a methotrexate-resistance gene (clinicaltrials.gov identifier NCT01683279). Several CD19 CAR trials are recruiting at Baylor including a Phase 1 trial that will study the simultaneous infusion of both 2^nd^ and 3^rd^ generation CARs in patients with Non-Hodgkin Lymphoma (NHL) or CLL (clinicaltrials.gov identifier NCT01853631). CARs targeting the CD19 antigen are leading the way in the clinic and insights gained from these trials will be invaluable for the entire field.

### Wilms tumor antigen 1

The Wilms tumor protein (WT-1) is a zinc-finger transcription factor that is important in normal cellular development and cell survival. In the human embryo, WT-1 is required for normal kidney development and, in adults, WT-1 has low expression levels in hematopoietic progenitor cells and in the ovary, testis, podocytes of the kidney, and peritoneal and pleural mesothelium [172, 173]. Alternative splicing generates several isoforms of WT-1 and depending upon the cell in which the gene is expressed, it can function as either an oncogene or a tumor suppressor [174]. WT-1 represents a TAA as it is overexpressed in both leukemias (AML, CML, myelodysplastic syndrome [MDS], and ALL) and various solid tumors (ovary, breast, renal cell, colon, lung), where it acts as a putative oncogene [175–179]. Up to 70% of AML cases overexpress WT-1, where it is a marker of poor prognosis and minimal residual disease [180–183]. Although its precise biologic role in leukemia remains unknown, Yamagami et al. demonstrated that knockdown of WT-1 via antisense oligomers significantly inhibited the growth of primary leukemic clones, suggesting that the protein plays an important role in leukemogenesis [184]. Thus, WT-1 represents an attractive immunotherapeutic target, and there are currently 3 open clinical trials for adult leukemia patients that center around this unique antigen.

There are numerous preclinical studies that have demonstrated diverse and feasible approaches to target WT-1 in the clinic, and these have been expertly reviewed by O’Reilly et al. from MSKCC [185]. Briefly, several groups have demonstrated that WT-1-specific, functional CD4 and CD8 T-cells can be generated from healthy donor PBMC by stimulation with various WT-1 peptides [186–190]. Spontaneous autologous T-cell responses against WT-1 can also be detected in the peripheral blood of leukemia patients, providing rationale for either allogeneic or autologous ACT approaches [191, 192]. More recently, Xue et al. have engineered a retroviral vector encoding the T-cell receptor alpha and beta chains derived from a WT-1 TCR specific for the HLA-A0201-restricted RMF peptide. This TCR construct was optimized by incorporating a disulfide bond to prevent alpha/beta mispairing and, when transduced into a CML patient’s T-cells, mediated prevention of autologous leukemia engraftment in an immunocompromised mouse model [100, 193]. This TCR is now in clinical testing in London (see below). To broaden WT-1 specific ACT to patients with less common HLA subtypes, investigators have developed an elegant system in which donor T-cells are stimulated with pools of overlapping WT-1 peptides generating WT-1 specific T-cells with specificities that can be decoded using IFN-gamma secretion and a matrix of known peptide sequences. Using this approach T-cells specific for 27 different WT-1 peptides with diverse HLA-restriction were shown to lyse WT-1+ leukemic targets [194]. This approach, too, is now being tested in the clinic. As a final mention in the preclinical realm, Dao et al. have developed a full-length humanized TCR-mimic antibody from a phage display library that is specific for the WT-1 RMF peptide in the context of HLA-A0201. This antibody (ESK1) was shown to be highly avid and in 2 different mouse models, nearly ablated leukemia xenografts from 2 ALL cell lines, both alone and in concert with adoptive transfer of human effector cells [115]. The scFv from an affinity matured WT-1 TCR-mimic antibody has since been integrated into a CAR construct that has demonstrated lysis of solid tumor and B-cell leukemia cell lines [67].

As of June 2014, the clinicaltrials.gov website lists 3 Phase 1/2 trials that are using ACT to target the WT-1 antigen in leukemia patients. The first is based at University College in London and uses the retroviral vector developed by Xue et al. (discussed above) to redirect autologous T-cells from HLA-A0201-positive CML and AML patients via coexpression of a TCR specific for the A2-restricted WT-1 RMF peptide (clinicaltrials.gov identifier NCT01621724). This group hypothesizes that transducing and infusing fresh autologous, engineered T-cells will result in greater persistence of the WT-1 redirected cells compared to those from long-term culture. The second trial is sponsored by MSKCC and builds upon their success in generating functional, WT-1 specific T-cells from healthy donors with diverse HLA subtypes by *in vitro* stimulation with APCs pulsed with pools of overlapping WT-1 peptides [185, 194]. This trial includes patients with AML, MDS, CML or ALL who are at high risk of relapse after alloSCT and utilizes infusions of WT-1-specific donor T-cells that are expanded *in vitro*, cryopreserved, and then infused at the time of recurrence or appearance of MRD. Reportedly, these infusions have been well tolerated without renal, hematopoietic, or GvHD toxicity, and they can transiently reduce or eliminate WT-1-expressing cells from the circulation [185] (clinicaltrials.gov identifier NCT00620633). The third trial is based at the Fred Hutchinson Cancer Research Center in Seattle and builds upon this center’s success in an earlier pilot trial. In the pilot trial, 11 HLA-A201-positive patients with AML or ALL who had received alloSCT were treated with adoptive transfer of donor-derived, *in vitro* expanded WT-1 RMF peptide-specific CD8+ T-cells. Here, the addition of the cytokine IL-21 to the *ex vivo* T-cell expansion protocol for the last 4 patients resulted in improved persistence of the WT-1-specific T-cells and expression of phenotypic markers associated with long-lived memory [25]. The infusions of large numbers of WT-1 specific T-cells (up to 1 × 10^10^ cells/m^2^) were safe and no GvHD, hematologic, or renal toxicity was observed. Encouragingly, of the 4 patients treated with antigen-specific T-cells generated in the presence of IL-21, all 4 experienced persistence of the WT-1 specific T-cells and demonstrated durable CRs (22–38 months post SCT) despite a ~90-95% risk of recurrence, though 1 patient has since relapsed. In the current Phase 1/2 trial, a TCR isolated from a very high avidity T-cell clone specific for the A2-restricted RMF WT-1 peptide has been isolated and is being retrovirally transduced into donor T-cells (similar to the London trial except in an allogeneic setting). In this trial, eligible patients are those with AML, CML, or MDS who are post-SCT, HLA-A0201-positive, and are at high-risk of relapse or who recur with overt or MRD positive disease. These patients will be treated with multiple infusions of donor EBV or CMV-specific T-cells that are transduced to coexpress the WT-1 specific TCR. The rationale for transducing only virus-specific T-cells is that in the allogeneic setting, transduction of T-cells with unknown secondary specificities would likely result in considerable GvHD. Patients will also receive IL-2 infusions to boost persistence of the antigen-specific cells.

### Lewis Y

The Lewis Y antigen (Le^Y^) is a difucosylated carbohydrate present on various cell surface proteins and lipids [195]. It is related to the Lewis blood group of antigens but is not expressed on red blood cells. In normal tissues, the Le^Y^ antigen shows low levels of surface expression on the gastrointestinal mucosa, ciliated epithelium of the trachea and bronchus, and on neutrophils [196, 197]. It is primarily studied as a potential tumor-associated antigen (TAA) in the context of solid malignancies since the Le^Y^ antigen is highly expressed in 60-90% of epithelial cancers including those of the breast, pancreas, ovary, colon, stomach and lung [198, 199]. A recent study also showed expression of the Lewis Y antigen in ~ 50% of AML and multiple myeloma (MM) cases, suggesting it could be a targetable TAA in a wide range of malignancies [200]. Its biologic function remains unknown though higher expression of Le^Y^ correlates with poorer prognosis in lung cancer, suggesting a potential role for Lewis Y in maintaining a malignant phenotype [201].

Preclinical validation of the Le^Y^ antigen gained momentum when Kitamura et al. developed an anti-Le^Y^ humanized monoclonal antibody – known as Hu3S193 [202]. This antibody was used alone and as an immunoconjugate in several early phase clinical trials targeting the Lewis Y antigen in patients, first in those with solid malignancies. The humanized monoclonal antibody Hu3S193 (also used to generate the anti-Le^Y^ CAR discussed below) was tested in a Phase I trial in 15 patients with primarily breast, colorectal, or lung cancer. There were no objective tumor responses, however, there was significant uptake of the antibody in tumor metastases and no uptake in normal tissue. Critically, the antibody infusion was safe with only one Grade 3 or 4 adverse effect - an elevated alkaline phosphatase in a patient with extensive hepatic metastases [203]. In an effort to increase the potency of tumor response, two Phase I trials were conducted using the Lewis Y antibody, Hu3S193, conjugated to either doxorubicin or calicheamicin. In the Hu3S193-doxorubicin immunoconjugate trial, 66 patients with mainly metastatic colon or breast cancer were treated, and significant, dose-limiting GI toxicity was observed, likely due to Lewis Y expression on normal GI epithelium. Objective responses were seen in only 2 patients [204]. In the Hu3S193-calicheamicin immunoconjugate trial, 9 patients with solid tumors were enrolled. Disappointingly, this conjugate showed primary localization to the liver instead of tumor and demonstrated rapid hepatic clearance from the circulation [205].

The promising yet limited effects of the Hu3S193 antibody laid the foundation for the targeting of Le^Y^ using ACT. In preclinical studies, Hu3S193 was used to generate a 2^nd^ generation anti-Le^Y^ CAR, which was first tested against various malignant epithelial-derived cell lines and in a mouse xenograft model of ovarian cancer. The anti-Le^Y^ CAR T-cells lysed breast and colorectal cell lines *in vitro*, and lysis correlated positively with the level of Le^Y^ expression. Importantly, no IFN-γ release or lysis was observed against neutrophils, which have been shown to express Lewis Y at low levels. Further, in immunocompromised mice anti- Le^Y^ CAR T-cells mediated regression of established human ovarian tumors, though tumors did eventually recur [198]. More recently, this same CAR was used in preclinical testing against AML and MM, which also show overexpression of the Lewis Y antigen. The CAR T-cells lysed human MM and AML cell lines *in vitro* and delayed the incidence of plasmacytomas in a NOD/SCID mouse model, though no lysis of primary malignant cells or rejection of established myeloma or AML was demonstrated [200].

The first and (thus far) only ACT clinical trial targeting the Le^Y^ antigen in patients utilized the 2^nd^ generation anti-Le^Y^ CAR mentioned above, which demonstrated activity against both solid and hematologic tumors in preclinical studies. Results from the first 5 enrolled patients, all with AML, were recently published and are summarized here [43]. This Phase I trial enrolled patients with high-risk or relapsed/refractory AML or MM whose tumors demonstrated expression of Le^Y^ on at least 20% of blasts, with a median fluorescence intensity twice that of normal lymphocytes. It is worth noting that the first 5 patients enrolled had relatively low Lewis Y expression compared to the patient samples used in the study that first described the presence of Lewis Y antigen in AML and MM [200]. Three of the four patients who underwent CAR T-cell infusion (one patient died from induction chemotherapy) had cytogenetic MRD prior to CAR T-cell infusion; the fourth patient had 70% blasts in the bone marrow as well as circulating disease. Patients were preconditioned with fludarabine and cytarabine and then infused with a median of 4.45 × 10^6^ anti-Le^Y^ CAR T-cells/kg. As a comparison, these cell doses are comparable to those administered in the successful CD19 CAR clinical trials [134]. There were no grade 3 or 4 toxicities, and there was evidence of biologic activity though responses were modest at best. Of the 3 patients with MRD prior to CAR T-cell infusion, 1 demonstrated a sustained morphologic remission of 23 months though cytogenetic clones remained detectable, the 2^nd^ patient showed a transient cytogenetic remission of 5 months, and the 3^rd^ patient did not respond and relapsed 49 days after infusion. The 4^th^ patient who had significant disease prior to anti-Le^Y^ CAR treatment had a very transient reduction in blasts, though the effect attributable to the CAR cells in the context of pretreatment with fludarabine and cytarabine is difficult to discern. Encouragingly, the CAR T-cells did show trafficking to the bone marrow and persisted in 3 of the 4 patients for 2, 4 and 10 months, though with variable transgene copy number by qualitative PCR. Perhaps most disappointingly, the 3 patients who showed some biologic response all relapsed with myeloblasts that still expressed the Lewis Y antigen with comparable MFIs to their pre-CAR treatment disease; there was no antigenic shift despite persistence of the CAR cells. The authors hypothesize that this could be due to an immunosuppressive microenvironment or downregulation in transgene expression. It might simply be related to the relatively low expression of Lewis Y on the myeloblasts and inadequate activation of the modified cells. The authors suggest the next anti-Le^Y^ CAR T-cell trial will be performed in lung cancer patients, where there is a more dense and uniform expression of the Le^Y^ antigen.

### κ Light chain

The major long-term toxicity associated with the successful anti-CD19 CAR T-cell trials discussed above has been B-cell aplasia, which is secondary to the expression of CD19 on normal and B-cell precursors [79, 80, 134, 135, 164]. A strategy to avoid this adverse effect has been developed using engineered 2^nd^ generation CAR T-cells with specificity for the κ light chain of surface immunoglobulin (sIg) within the B-cell receptor [206]. The concept behind this approach is that B-cell malignancies are clonal disorders wherein a patient’s malignant cells express a κ or λ-restricted sIg, but not both. In those patients with a κ-restricted malignancy, the anti-κ CAR T-cells would ideally eliminate all malignant cells and, consequently, normal B-cells with κ expression; however, normal λ-restricted B cells would be left untouched providing these patients with adequate B-cell immunity [207, 208].

In preclinical studies, the κ-light chain specific CAR T-cells selectively lysed κ-expressing lymphoma and leukemic cell lines as well as autologous and allogeneic κ-restricted primary CLL cells [206]. *In vivo*, human T-cells retrovirally transduced with the anti-κ CAR mediated regression of lymphoma xenografts. As an aside, this was among the first reports to show the importance of a CD28 costimulatory domain in the CAR construct, and it confirmed in this preclinical study that CD28 improved the expansion of the engineered anti-κ T-cells [206, 209]. A Phase I trial is currently recruiting patients with relapsed CLL, NHL, or MM to study treatment with autologous 2^nd^-generation anti-κ CAR T-cells (clinicaltrials.gov identifier NCT00881920). The trial will assess the safety and, as a secondary measure, the survival and function of anti-κ T-cells at 3 escalating T-cell doses after lymphodepletion with cyclophosphamide. It will be important to see whether antigen escape limits the success of this approach since MM and CLL are known to have variable expression of sIg [210–212]. Moreover, the antibody portion of the CAR construct is derived from a mouse, and the immunogenicity of this CAR in humans remains to be seen. Conversely and in support of this approach, targeting the B-cell receptor in lymphoma through idiotype vaccines has shown great promise and points to the feasibility of using the B-cell receptor (whether the idiotype or the light chain portion) as a TAA [41].

### HA-1 and other minor histocompatibility antigens

As defined above, minor histocompatibility antigens (mHAs) are peptides derived from endogenous, polymorphic proteins that differ between donor and recipient and are thought to account for much of the GvL effect within HLA-matched donor-recipient pairs [213]. These antigens can also trigger GvHD, and therefore the major goal with mHAs has been to identify and target mHAs that show predominant expression in the hematopoietic compartment. Strong preclinical evidence for targeting these mHAs was demonstrated in an immunocompetent mouse model where targeting a single mHA (B6^dom1^) resulted in complete eradication of leukemia without GvHD [214]. Two recent clinical trials have investigated unique approaches towards this common objective of treating leukemia by targeting hematopoietic-restricted mHAs.

In one clinical trial utilizing adoptive transfer of mHA-specific T-cells, investigators first designed an *in vitro* culture system to enrich for mHA-specific donor T-cells [215]. In this trial, PBMC were collected from patients at post-transplant timepoints when their hematopoietic system had been replaced by donor cells. These donor-derived cells were then stimulated *in vitro* with pre-transplant, irradiated recipient PBMC and recipient EBV-LCLs (Epstein Barr Virus-transformed B lymphoblastoid cell lines) in an effort to generate donor T-cells specific for hematopoietic mHAs [216]. CD8+ T-cell clones were then generated using limiting dilution cloning. Donor CD8+ clones that lysed recipient EBV-LCLs but neither donor EBV-LCLs nor recipient fibroblasts were selected for treatment of 7 patients with relapsed MDS or ALL after alloSCT. Patients received cytoreductive chemotherapy but not specific lymphodepletion prior to T-cell infusions, which were administered in a dose-escalation protocol. Unfortunately, most of the patients had GvHD prior to treatment so it was difficult to grade the degree of GvHD caused by these T-cells, though no cases were directly attributable to the clones. Surprisingly, the major toxicity observed was pulmonary toxicity, which occurred in 3 patients and underscores the reality that adoptively transferred T-cells tend to initially accumulate in the lungs making this a vulnerable site for adverse effects [217]. Five of 7 patients achieved transient CRs, and 3 of these responses were directly attributable to the CTL clones and not the cytoreductive chemotherapy. A genome-wide, single nucleotide polymorphism-based correlation was used to identify the mHA targeted by 3 of the generated clones - 2 that were novel [218]. Interestingly, mRNA expression analysis suggested that leukemic blasts downregulated expression of 1 of the targeted mHAs after CTL treatment, again illustrating that it may be important to target proteins directly involved in leukemogenesis. Two of the patients who demonstrated significant pulmonary toxicity were infused with clones that recognized epitopes from proteins also expressed on the pulmonary alveolar epithelium, and the CTLs were shown to be directly causative in these adverse reactions. The CTLs showed potent effector function but did not persist past 21 days, possibly as a result of their extended time in culture that resulted in a differentiated, effector population of CD8+ T-cells. Unfortunately, all patients who demonstrated an initial response relapsed and succumbed to their disease.

In a more recent Phase I study, Meij et al. tested the feasibility and safety of treating relapsed post-SCT AML and CML patients with donor-derived T-cells specific for the mHA, HA-1 [35, 219]. HA-1-specific T-cells were generated from healthy donor PBMC using donor dendritic cells pulsed with the HLA-A2 restricted HA-1 peptide during a 5-week *in vitro* culture period. The cells were then infused into patients in the absence of preconditioning or *in vivo* cytokine administration. The cells were primarily from the effector memory subset and 6-27% were tetramer positive (i.e. HA-1-specific). Unfortunately, no objective responses were demonstrated though a patient with CML did have stable disease for 3 months. HA-1 specific T-cells were detected only in this CML patient and were present for ~ 8 weeks. No GvHD was reported. The authors concluded that more potent responses might be generated by shortening the *in vitro* culture process possibly via ectopic TCR alpha/beta transfer and also through use of physiologic concentrations of cytokines during the *in vitro* expansion. Towards this goal, a codon optimized and cysteine modified HA-1-TCR α/β retroviral construct has been designed for use in clinical trials [108].

### BCR-ABL

Tumor-specific antigens (neoantigens) within the BCR-ABL1 fusion tyrosine kinase have also been investigated as targets for ACT of CML. Several groups have shown that CML patients have functional, circulating T-cells specific for these neoantigens that can be expanded with a peptide vaccine [39, 53, 220]. A recent clinical trial investigated the feasibility and safety of prophylactically administering donor CD8+ T-cells enriched for specificities against peptides from WT-1, PR1, and BCR-ABL to CML patients after T-cell depleted alloSCT [57]. Five patients were treated with cells enriched for CTL with specificity for HLA-A3, A11, or B8-restricted BCR-ABL neopeptides. In these patients, the cells infused were largely polyclonal DLIs with wide ranges in the percentage of BCR-ABL-specific cells actually administered – from 0.1 – 17%. Though few patients were enrolled and the effects of therapy attributable to ACT are indiscernible from those related to alloSCT, 1 patient who received large numbers of BCR-ABL specific donor T-cells that exhibited robust *in vitro* cytotoxicity has remained in molecular remission for > 40 months and antigen-specific cells have persisted at high frequencies for >1 year.

A separate report described the successful treatment of a CML patient with leukemia-reactive CTL in 1999, prior to the advent of many of the engineering techniques now available. In this study, the CML patient had relapsed with accelerated-phase disease after alloSCT and DLI and was treated with donor cells enriched for leukemia-specific antigens via multiple rounds of *in vitro* stimulation with patient leukemia cells. The T-cells were not fully characterized but potentially recognized neoepitopes from BCR-ABL among others. This patient experienced molecular remission for 2 years until she later died of ischemic heart disease [221]. These studies offer proof-of-principle for targeting leukemia neoantigens though larger scale studies are needed using modified T-cells or updated *in vitro* expansion protocols. Table 2 summarizes the antigens discussed in the section.

**Table 2:** **Summary of hematologic tumor antigens being targeted in clinical trials of ACT**

Antigen	HLA-restriction	Hematologic Malignancy ^†^	Immunotherapeutic Potential
CD19	No	ALL, CLL, Lymphoma	1) Common to several lymphoid malignancies
			2) Highly potent effectors have been developed
			3) Cell surface protein that does not require processing or MHC presentation
			4) Anti-leukemia effects must be balanced with toxicity against normal B cells
WT-1	Yes	AML, CML, MDS, ALL	1) Has been studied primarily in myeloid hematologic malignancies, although recent work suggests its potential in lymphoid malignancies [222]
			2) Minimal expression in normal tissues
			3) Requires intracellular processing and antigen presentation
Lewis Y	No	AML, MM	1) At this time, studies are limited to AML and MM
			2) Studies to date have shown moderate potency against malignant cells
			3) May be limited by GI toxicity
κ Light Chain	No	CLL, lymphoma, MM	1) Limited to lymphoid malignancies and MM
			2) Still in early stages of development, therefore potency is difficult to assess
mHA	Yes	Applicable to all hematologic malignancies	1) Potent immune responses against malignant cells, including stem cells
			2) May also trigger GvHD
			3) Limited by the frequency of the minor allele in the population
			4) Difficult to predict off-target tissue expression
BCR-ABL	Yes	CML	1) Limited studies using ACT to target BCR-ABL in the era of tyrosine kinase inhibitors
			2) Mainly useful in CML and possibly Ph^+^ ALL

^**†**^Expression of the antigens has been reported in other hematologic and solid tumor malignancies.

## Conclusions

In summary, ACT represents a promising strategy for treating leukemia patients. The list of antigens discussed above is by no means exhaustive and many ACT strategies are in the pipeline to target other leukemia antigens including PR1 [29], human telomerase reverse transcriptase (hTERT) [26], receptor tyrosine kinase-like orphan receptor 1 (ROR1) [45], CD23 [223], CD123 [224], preferentially expressed antigen in melanoma (PRAME) [27], hyaluronan-mediated motility receptor (HMMR/Rhamm) [28], CD22 [44], cathepsin G [30] and aurora kinase a (AURKA) [225] among others. The clinical success achieved using anti-CD19 CARs has shifted momentum in favor of this type of engineered receptor, but exciting clinical trials utilizing ectopic TCR α/β receptors and even conventional T-cells are also underway. Significant challenges remain in outlining the best setting for ACT of leukemia, though this will likely vary by disease type, targeted antigen, and T-cell approach. Moreover, incorporating automation and standardization will be critical to shifting the ACT field away from intricate protocols and small, single-center trials towards broader applicability. The goal is to harness and refine the tremendous specificity and potency of lymphocytes, best evidenced by the ability of alloSCT and DLI to cure otherwise incurable diseases (CML, CLL), into a T-cell treatment for leukemia that is more effective and less toxic than current standard of care therapies.

## Abbreviations

ALL: Acute lymphoblastic leukemia
AML: Acute myeloid leukemia
ACT: Adoptive cellular therapy
alloSCT: Allogeneic stem cell transplantation
APCs: Antigen presenting cells
CAR: Chimeric antigen receptor
CLL: Chronic lymphocytic leukemia
CML: Chronic myeloid leukemia
CR: Complete response
CMV: Cytomegalovirus
CTLs: Cytotoxic T-lymphocytes
DLI: Donor lymphocyte infusion
EBV: Epstein Barr Virus
GvHD: Graft-versus-host disease
GvL: Graft-versus-leukemia
HLA: Human leukocyte antigen
MHC: Major histocompatibility complex
MRD: Minimal residual disease
mHA: Minor histocompatibility antigen
MM: Multiple myeloma
MDS: Myelodysplastic syndrome
NK cell: Natural killer cell
NHL: Non-Hodgkin Lymphoma
PR: Partial response
PBMCs: Peripheral blood mononuclear cells
PTLD: Post-allogeneic stem cell transplant lymphoproliferative disease
scFv: Single chain variable fragment
sIg: Surface immunoglobulin
TCR: T-cell receptor
TAA: Tumor-associated antigen
TIL: Tumor infiltrating lymphocyte
WT-1: Wilms tumor antigen.
